# Aromaticity and Antiaromaticity Reversals between the Electronic Ground State and the Two Lowest Triplet States of Thiophene

**DOI:** 10.1002/cphc.202400758

**Published:** 2024-11-05

**Authors:** Edward Cummings, Peter B. Karadakov

**Affiliations:** ^1^ Department of Chemistry University of York Heslington, York YO10 5DD UK

**Keywords:** Excited state aromaticity reversals, Nucleus-independent chemical shift, Off-nucleus shielding, Baird's rule, Thiophene

## Abstract

It is shown, by examining the variations in off‐nucleus isotropic magnetic shielding around a molecule, that thiophene which is aromatic in its electronic ground state (S_0_) becomes antiaromatic in its lowest triplet state (T_1_) and then reverts to being aromatic in T_2_. Geometry relaxation has an opposite effect on the aromaticities of the ππ* vertical T_1_ and T_2_: The antiaromaticity of T_1_ is reduced whereas the aromaticity of T_2_ is enhanced. The shielding picture around T_2_ is found to closely resemble those around certain second singlet ππ* excited states (S_2_), for example, those of benzene and cyclooctatetraene, thought to be “strongly aromatic” because of their very negative nucleus‐independent chemical shift (NICS) values. It is argued that while NICS values correctly follow the changes in aromaticity along the potential energy surface of a single electronic state, the use of NICS values for the purpose of quantitative comparisons between the aromaticities of different electronic states cannot be justified theoretically and should be avoided. “Strongly aromatic” S_2_ and T_2_ states should be referred to simply as “aromatic” because detailed comparisons between the properties of these states and those of the corresponding S_0_ states do not suggest higher levels of aromaticity.

## Graphical abstract

Thiophene is the first example of a molecule with a second triplet ππ* excited state (T_2_) which exhibits aromaticity levels similar to those observed in the second singlet ππ* excited state (S_2_) of benzene. However, even though the T_2_ state of thiophene is undeniably aromatic, with very negative nucleus‐independent chemical shift (NICS) values, a detailed analysis of its properties shows that it is not more aromatic than the ground state (S_0_).

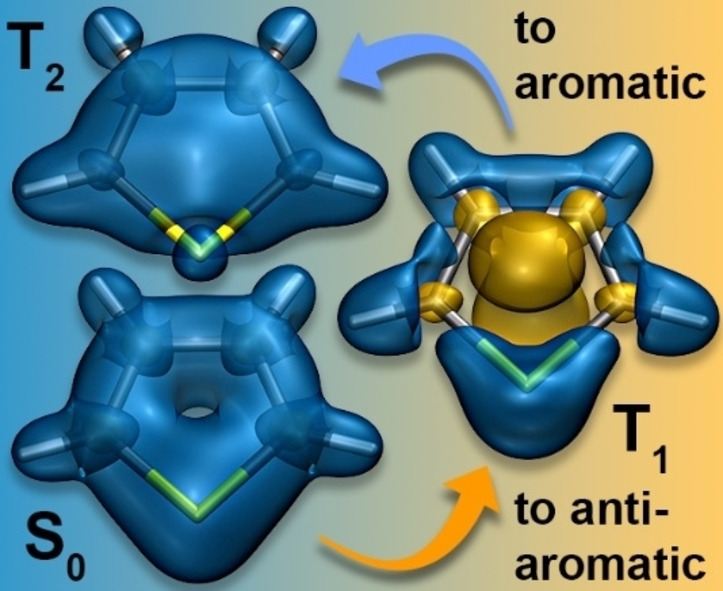

## Introduction

The formulation of Baird's rule^[1]^ marks the starting point of focused research into excited state aromaticity and antiaromaticity reversals. In addition to presenting a challenge to theoretical chemists, these phenomena have a number of applications including designing molecules with light‐controllable behaviour, molecular photoswitches,^[2]^ molecular motors,[^3^, ^4^] “flapping” fluorophores,[^5^, ^6^, ^7^] and rationalizing experimental evidence about photochemical reactions, for example, excited‐state intramolecular proton transfers[^8^, ^9^]; further aspects of excited state aromaticity reversals have been discussed in several reviews.[^10^, ^11^, ^12^] According to Baird's rule, the familiar Hückel 4*n*+2 and 4*n* rules for electronic ground state (S_0_) aromaticity in cyclic conjugated hydrocarbons are reversed in the lowest triplet ππ* state (usually T_1_): Rings with 4*n* π electrons attain aromatic character, whereas those with 4*n*+2 π electrons attain antiaromatic character. Similar aromaticity reversals have been shown to occur between S_0_ and the lowest singlet ππ* excited state (usually S_1_).[^13^, ^14^, ^15^, ^16^]

Other low‐lying electronic states of cyclic conjugated hydrocarbons can also exhibit magnetic properties such as nucleus‐independent chemical shifts (NICS)[^17^, ^18^, ^19^] and variations of the off‐nucleus isotropic magnetic shielding, *σ*_iso_(**r**)=1/3[*σ_xx_* (**r**)+*σ_yy_* (**r**)+*σ_zz_* (**r**)], within molecular space, that suggest certain levels of aromaticity or antiaromaticity. Calculations utilizing state‐optimized full π space complete‐active‐space self‐consistent field wavefunctions involving gauge‐including atomic orbitals (CASSCF‐GIAO) have shown that the second singlet excited states (S_2_) of benzene (1 ^1^B_2g_, *D*_6h_ point group),^[16]^ regular octagonal cyclooctatetraene (1 ^1^B_2g_, *D*_8h_ point group)^[20]^ and naphthalene (1 ^1^B_2u_, *D*_2h_ point group),^[21]^ corresponding to vertical excitations, exhibit NICS values that are significantly more negative than those for the S_0_ state of benzene; the interior of the carbon framework in these states is well‐shielded all over, but the carbon‐hydrogen bonds are less shielded than they are in the corresponding S_0_ states. While it is tempting to classify these S_2_ states as “strongly aromatic”, it is questionable whether, on the basis of comparisons between NICS values, it would be justified to assume that these S_2_ states are more aromatic than the S_0_ state of benzene. At the moment, state‐optimized CASSCF‐GIAO is the only theoretical approach capable of calculating magnetic shielding tensors for singlet excited states and these findings have not been confirmed with an alternative theoretical method; there have been no theoretical results suggesting the existence of similar “strongly aromatic” triplet states. It should be noted that state‐optimized full π space CASSCF‐GIAO calculations on anthracene (*D*_2h_ point group) also predict a “strongly aromatic” vertical singlet excited state, however, in contrast to naphthalene, this state is S_1_ (1 ^1^B_2u_), whereas the lowest singlet antiaromatic state is S_2_ (1 ^1^B_3u_).^[21]^ The aromaticities of the vertical S_1_ and S_2_ states of higher acenes are expected to follow the pattern observed in anthracene.

Most of the research on excited state aromaticity and antiaromaticity reported in the literature targets the T_1_ state which is much easier to access computationally than the S_1_ state through spin‐unrestricted density functional theory (UDFT) calculations. While time‐dependent DFT (TD‐DFT) and TD‐DFT employing the Tamm‐Dancoff approximation (TDA‐DFT) can be used to calculate excitation energies and optimize excited state geometries, at the moment, there are no codes allowing calculations of magnetic shielding tensors using these methods.

In this paper we investigate the aromaticity reversals between the S_0_, T_1_ and T_2_ states of thiophene using DFT, UDFT, TD‐DFT and TDA‐DFT to calculate excitation energies and optimize ground and excited state geometries, and DFT‐GIAO and UDFT‐GIAO to calculate magnetic shielding tensors for all three states. We show that while, as expected, the S_0_ state of thiophene is aromatic and the vertical T_1_ state is antiaromatic,^[22]^ the magnetic properties of the vertical T_2_ state closely resemble those of the “strongly aromatic” vertical S_2_ states of benzene, regular octagonal cyclooctatetraene and naphthalene. The geometry optimizations of the antiaromatic vertical T_1_ state and of the “strongly aromatic” vertical T_2_ state produce non‐planar geometries with reduced antiaromaticity and enhanced aromaticity, respectively.

Our results demonstrate that the magnetic properties of the “strongly aromatic” low‐lying vertical singlet excited states observed so far can also be exhibited by low‐lying vertical triplet excited states. This is a strong indication that these properties are not a computational artefact of CASSCF‐GIAO calculations but characterize states in which the shielding activity of a subset of electrons is concentrated within the ring interiors and gives rise to very negative NICS values. However, on the basis of these very negative NICS values only, it is not reasonable to expect that the overall levels of aromatic stabilization for such states will be higher than those for aromatic S_0_ states. We argue that, whereas changes in the signs of NICS values can be used to identify excited state aromaticity reversals, comparisons between the magnitudes of NICS values of the same sign for different electronic states are unlikely to provide reliable information about relative levels of aromaticity and should be avoided.

## Computational Details

All geometry optimizations, analytical harmonic frequency and shielding calculations reported in this paper were carried out in the gas phase using GAUSSIAN.^[23]^ The optimized geometries were confirmed as local minima through analytical harmonic frequency calculations. The S_0_ (1 ^1^A_1_) geometry of thiophene (*C*_2v_ symmetry) was optimized using B3LYP‐D3(BJ) (spin‐restricted B3LYP with Grimme's D3 empirical dispersion corrections and Becke–Johnson damping), with two basis sets, def2‐TZVP and def2‐TZVPPD. The def2‐TZVPPD basis sets for C, H and S are not included in the GAUSSIAN basis set library and were obtained from the Basis Set Exchange.^[24]^

Accidentally, we observed that the initial guesses for the Kohn‐Sham orbitals in triplet UB3LYP/6‐311++G(d,p) and UB3LYP/6‐311++G(2d,2p) calculations at the S_0_ B3LYP‐D3(BJ)/def2‐TZVP geometry were of different symmetries, B_2_ and A_1_, respectively, and then the self‐consistent field (SCF) procedure “locked” on these symmetries and converged to the corresponding vertical triplet states, T_1_ (1 ^3^B_2_) and T_2_ (1 ^3^A_1_). Analogous behaviour was observed at the S_0_ B3LYP‐D3(BJ)/def2‐TZVPPD geometry. Use of the Kohn‐Sham orbitals from the triplet UB3LYP/6‐311++G(d,p) calculation as the initial guess allowed triplet UB3LYP calculations utilizing the 6‐311++G(2d,2p), def2‐TZVP and def2‐TZVPPD basis sets to converge to the vertical T_1_ state; triplet UB3LYP calculations utilizing these three basis sets and the default GAUSSIAN initial guess converge to the vertical T_2_ state. These observations are specific for thiophene and there is no guarantee that a similar procedure would work for other cyclic conjugated molecules.

S_0_→T_1_ and S_0_→T_2_ vertical excitation energies were calculated using UB3LYP, TD‐B3LYP and TDA‐B3LYP and the def2‐TZVP and def2‐TZVPPD basis sets, at the S_0_ B3LYP‐D3(BJ)/def2‐TZVP and B3LYP‐D3(BJ)/def2‐TZVPPD geometries, respectively. The geometries of the T_1_ and T_2_ states of thiophene were optimized using UB3LYP‐D3(BJ) and TDA‐B3LYP‐D3(BJ), with the def2‐TZVP and def2‐TZVPPD basis sets.

NICS values and volume data required to construct the S_0_ isotropic shielding isosurface were obtained through B3LYP‐GIAO/6‐311++G(2d,2p) calculations at the B3LYP‐D3(BJ)/def2‐TZVPPD optimized geometry of thiophene. NICS values and volume data needed for the T_1_ and T_2_ isotropic shielding isosurfaces were obtained in a similar way, using UB3LYP‐GIAO/6‐311++G(2d,2p) calculations at the S_0_ B3LYP‐D3(BJ)/def2‐TZVPPD optimized geometry for vertical excitations, or at the respective T_1_ and T_2_ UB3LYP‐D3(BJ)/def2‐TZVPPD optimized geometries. In all volume data calculations *σ*_iso_(**r**) was evaluated at regular three‐dimensional grids of points with a spacing of 0.05 Å. To reduce computational effort, shielding tensors were calculated at the symmetry‐unique points and then data was replicated through symmetry operations to create a complete grid. To enable visualization, the *σ*_iso_(**r**) volume data for each of the following five states of thiophene: S_0_, vertical T_1_ and T_2_, and T_1_ and T_2_ at the respective optimized geometries, were assembled in a GAUSSIAN cube file. *σ*_iso_(**r**) contour plots were constructed by extracting the required data from the three‐dimensional grids.

NICS(0)^[17]^ and NICS(1)[^18^, ^19^] values at the planar S_0_ geometry of thiophene were calculated according to the standard definitions, as −*σ*_iso_(at the ring center) and −*σ*_iso_(at 1 Å above the ring center), respectively. The optimized geometries of the T_1_ and T_2_ states of thiophene are non‐planar. The NICS(0) positions at these geometries were chosen as the averages of the coordinates of the sulfur and carbon atoms; in order to calculate NICS(±1) values, a plane was fitted to the coordinates of the ring atoms and the NICS(0) position, and the NICS(±1) positions were taken as the points 1 Å above and below that plane along the normal passing through the NICS(0) position following the procedure outlined in refs. [25–26].

As usual in NICS and ring current calculations on triplet systems (see, for example, refs. [27,28]) the UB3LYP‐GIAO magnetic properties of the triplet states of thiophene computed in this paper include the contributions arising from the perturbation to the Kohn‐Sham orbitals only. The omission of the large terms arising from the interaction of the electronic spin angular momentum with the magnetic field[^29^, ^30^] implies that the reported numbers will exhibit considerable differences from experimental measurements if and when such measurements become available. The advantage of the approach adopted here is that the values reported for triplet states can be compared directly to those for singlet states.

HOMA (harmonic oscillator model of aromaticity)[^31^, ^32^, ^33^] calculations were performed with Multiwfn^[34]^ using the default parametrization. The decision to use the default parametrization rather than HOMER, a reparameterization of HOMA for T_1_ excited states,^[35]^ is associated with the need to compare the aromaticity levels at the optimized S_0_, T_1_ and T_2_ geometries of thiophene. In addition, the HOMER parameters published in ref. [35] cover C−C, C−N, N−N and C−O bonds only, and the determination of parameters for C−S bonds would require the selection of suitable reference molecules and a significant volume of further computational work.

GAUSSIAN cube files with shielding data for the S_0_, T_1_ and T_2_ states of thiophene, detailed optimized geometries of these states, additional computational details, spin‐density distributions around the T_1_ and T_2_ states and cartesian coordinates for all geometries optimized using the def2‐TZVPPD basis set and the corresponding energies, lowest vibrational frequences and <*S* ^2^> values for UB3LYP calculations are included in the Supporting Information. The *σ*_iso_(**r**) volume data provided in the GAUSSIAN cube files allow inspection of various aspects of the shielding distributions around the S_0_, T_1_ and T_2_ states of thiophene, including the construction of shielding isosurfaces at different *σ*_iso_(**r**) values, *σ*_iso_(**r**) contour plots in various planes and *σ*_iso_(**r**) scans along various directions.

## Results and Discussion

The S_0_ B3LYP‐D3(BJ) thiophene geometries obtained with the def2‐TZVP and def2‐TZVPPD basis sets turned out to be very similar, and in very good agreement with the optimized geometry from a coupled‐cluster calculation with single, double, and perturbative triple excitations in the cc‐pCV5Z basis set [CCSD(T)/cc‐pCV5Z],^[36]^ as well as with a precise semi‐experimental equilibrium structure.^[36]^ Selected structural parameters from the S_0_ B3LYP‐D3(BJ)/def2‐TZVPPD optimized geometry are shown in Table 1. The only more noticeable difference between the current S_0_ B3LYP‐D3(BJ) optimized geometries and the precise semi‐experimental equilibrium structure^[36]^ is in the carbon‐sulfur bond length which is overestimated by B3LYP‐D3(BJ) by about 0.008 Å (see Table S1).

**Table 1:** Structural parameters from the S_0_, T_1_ and T_2_ geometries of thiophene optimized using the def2‐TZVPPD basis set (bond lengths in Å, angles in degrees, for the atom numbering scheme, see Figure 1).

Parameter/Method	S_0_ B3LYP‐D3(BJ)	T_1_ UB3LYP‐D3(BJ)	T_1_ TDA‐B3LYP‐D3(BJ)	T_2_ UB3LYP‐D3(BJ)	T_2_ TDA‐B3LYP‐D3(BJ)
*R*(S_1_−C_2_)	1.718	1.771	1.770	1.766	1.769
*R*(C_2_−C_3_)	1.365	1.463	1.458	1.401	1.398
*R*(C_3_−C_4_)	1.422	1.343	1.348	1.435	1.439
*R*(C_2_−H)	1.077	1.081	1.080	1.076	1.076
*R*(C_3_−H)	1.080	1.080	1.080	1.080	1.080
∠(C_2_−S_1_−C_5_)	91.9	89.5	89.3	93.5	93.3
∠(S_1_−C_2_−C_3_)	111.5	111.1	111.6	105.6	105.5
∠(S_1_−C_2_−H)	120.3	120.2	120.3	118.0	118.1
∠(C_2_−C_3_−H)	123.4	121.8	121.9	123.2	123.3
∠(S_1_−C_1_−C_2_−C_3_)	0.0	9.3	8.4	17.9	18.3
∠(C_4_−C_3_−C_2_−H)	180.0	−147.3	−151.2	162.8	163.0
∠(C_5_−C_4_−C_3_−H)	180.0	−178.8	−178.7	−178.3	−178.5

The S_0_→T_1_ and S_0_→T_2_ vertical excitation energies calculated using UB3LYP, TD‐B3LYP and TDA‐B3LYP and the def2‐TZVPPD basis set at the S_0_ B3LYP‐D3(BJ)/def2‐TZVPPD geometry are shown in Table 2. The differences between these results and the corresponding results obtained with the def2‐TZVP basis set at the S_0_ B3LYP‐D3(BJ)/def2‐TZVP geometry are small, and we report only the results obtained with the def2‐TZVPPD basis set which are in slightly better agreement with the experimental data included in Table 2. Table 2 also includes a selection of computational results obtained by other authors (for other computational studies of the excited states of thiophene, see refs. [37–38]). Clearly, the TDA‐B3LYP vertical excitation energies are closer to the experimental band maxima than their TD‐B3LYP counterparts which is not unexpected in view of the conclusions following from more detailed comparisons between the performances of the two methods.^[39]^ The UB3LYP and TDA‐B3LYP S_0_→T_1_ vertical excitation energies are very close to the corresponding CASSCF estimate; however, the PT2F variant of multiconfigurational second‐order perturbation theory with a CASSCF reference (CASPT2) provides slightly better agreement with the experimental data. On the other hand, the UB3LYP and TDA‐B3LYP S_0_→T_2_ vertical excitation energies get a little closer to the experimental band maximum than PT2F. The DFT/MRCI (BHLYP combined with multi‐reference configuration interaction) vertical excitation energies are comparable to the current TD‐B3LYP results. According to the current TD‐B3LYP and TDA‐B3LYP results, the vertical T_1_ state is dominated by the π_3_→π_4_* orbital excitation with the next (much smaller) contribution coming from π_2_→π_5_*; the vertical T_2_ state is dominated by the π_2_→π_4_* orbital excitation with the next (much smaller) contribution coming from π_3_→π_5_*. This is in agreement with the findings of ref. [40] and the general expectation about the ππ* nature of the low‐lying excited states in heterocycles with five‐membered rings.^[41]^

**Table 2:** Vertical T_1_ (**1** ^3^B_2_) and T_2_ (1 ^3^A_1_) excitation energies of thiophene (in eV).

State	UB3LYP^[a]^	TD B3LYP^[a]^	TDA B3LYP^[a]^	DFT/MRCI^[40]^	CASSCF^[42]^	PT2F^[42]^	Exp. band maximum
T_1_	3.86	3.56	3.82	3.53	3.83	3.75	3.7,^[43]^ 3.75,[^44^, ^45^] 3.74^[46]^
T_2_	4.57	4.45	4.53	4.35	4.90	4.50	4.6,^[43]^ 4.62[^44^, ^45^, ^46^]

^[a]^ Results obtained with the def2‐TZVPPD basis set at the S_0_ B3LYP‐D3(BJ)/def2‐TZVPPD optimized geometry.

Considering the very reasonable agreement between the UB3LYP and TDA‐B3LYP S_0_→T_1_ and S_0_→T_2_ vertical excitation energies and the experimental data, we decided to use these two methods, with D3(BJ) corrections, for the optimizations of the geometries of the T_1_ and T_2_ states. The optimized geometries of each state obtained with the def2‐TZVP and def2‐TZVPPD basis sets are very similar, and so are the geometries of each state optimized with the UB3LYP‐D3(BJ) and TDA‐B3LYP‐D3(BJ) methods (see Tables S2 and S3 for detailed comparisons). Selected structural parameters from the T_1_ and T_2_ UB3LYP‐D3(BJ)/def2‐TZVPPD and TDA‐B3LYP‐D3(BJ)/def2‐TZVPPD optimized geometries are shown in Table 1. The current T_1_ and T_2_ optimized geometries are in good agreement with the UB3LYP T_1_ and TD‐B3LYP T_2_ optimized geometries reported by Marian et al.^[40]^

The *C*_2v_ symmetry of the S_0_ optimized geometry of thiophene is reduced to *C*_s_ in the optimized geometries of the T_1_ and T_2_ states; this is accompanied by a puckering of the thiophene ring along the C_2_−C_5_ line and by an out‐of‐plane bending of the hydrogen atoms attached to these carbon atoms, in an opposite direction from the sulfur atom in T_1_, and in the same direction as the sulfur atom in T_2_. The symmetries of the UB3LYP Kohn‐Sham determinants for the optimized T_1_ and T_2_ geometries are ^3^ A*′′* and ^3^ A*′*, respectively; these coincide with the symmetries of the TDA‐B3LYP descriptions of the T_1_ and T_2_ states at the respective optimized geometries. At the TDA‐B3LYP T_1_ optimized geometry the symmetries of the HOMO–1, HOMO and LUMO are *a′*, *a′′* and *a′*, respectively; T_1_ is dominated by the HOMO→LUMO orbital excitation, and T_2_ is dominated by HOMO–1→LUMO. At the TDA‐B3LYP T_2_ optimized geometry the symmetries of the HOMO–1, HOMO and LUMO are *a′′*, *a′* and *a′*, respectively; T_1_ is dominated by the HOMO–1→LUMO orbital excitation, and T_2_ is dominated by HOMO→LUMO.

**Figure 1:**
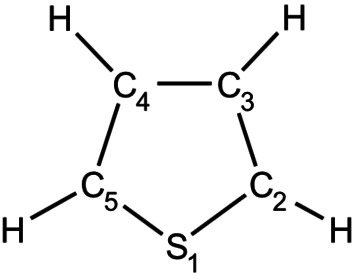
Atom numbering scheme for thiophene.

The carbon‐carbon bond alternation pattern changes between the S_0_ and T_1_ geometries: The C_2_−C_3_ and C_4_−C_5_ “double” bonds in S_0_ become “single” bonds in T_1_, and the C_3_−C_4_ “single” bond in S_0_ becomes a “double” bond in T_1_ Figure 1.

On the other hand, the T_2_ geometry exhibits a carbon‐carbon bond alternation pattern analogous to that in S_0_, albeit with longer carbon‐carbon bonds. These observations suggest that we could expect to observe some similarity between the aromaticities of the S_0_ and T_2_ states of thiophene, and possible aromaticity reversals between S_0_ and T_1_, as well as between T_1_ and T_2_. This expectation is confirmed by the HOMA values for the S_0_, T_1_ and T_2_ geometries of thiophene optimized using different methods and basis sets which are shown in Table 3. According to these HOMA values, thiophene is aromatic in S_0_, becomes mostly non‐aromatic in T_1_ and then reverts to being aromatic in T_2_, but less so than in S_0_. The small differences between the HOMA values for geometries optimized with the def2‐TZVP and def2‐TZVPPD basis sets highlight the similarity between these geometries; analogous observations can be made for the pairs of HOMA values for the T_1_ UB3LYP‐D3(BJ) and TDA‐B3LYP‐D3(BJ) geometries, and for the corresponding pairs of HOMA values for the T_2_ geometries. We note that HOMA cannot be used to examine the aromaticity changes associated with vertical excitations which utilize the S_0_ geometry.

**Table 3:** HOMA values for the S_0_, T_1_ and T_2_ geometries of thiophene optimized using the def2‐TZVP and def2‐TZVPPD basis sets.

Basis set	S_0_ B3LYP‐D3(BJ)	T_1_ UB3LYP‐D3(BJ)	T_1_ TDA‐B3LYP‐D3(BJ)	T_2_ UB3LYP‐D3(BJ)	T_2_ TDA‐B3LYP‐D3(BJ)
def2‐TZVP	0.822	−0.022	0.077	0.559	0.526
def2‐TZVPPD	0.821	−0.014	0.084	0.570	0.538

**Figure 2:**
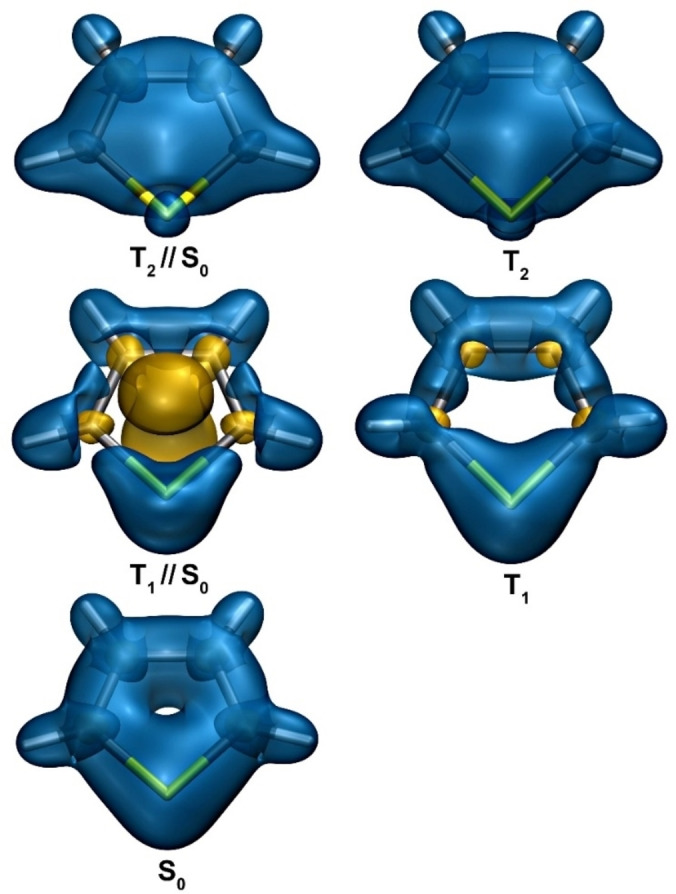
Isotropic shielding isosurfaces at *σ*_iso_(**r**)=±16 ppm (“+” in blue, “−” in orange) for the S_0_, T_1_ and T_2_ electronic states of thiophene. The T_1_//S_0_ and T_2_//S_0_ isosurfaces correspond to the S_0_→T_1_ and S_0_→T_2_ vertical excitations, respectively. For details, see text.

**Figure 3:**
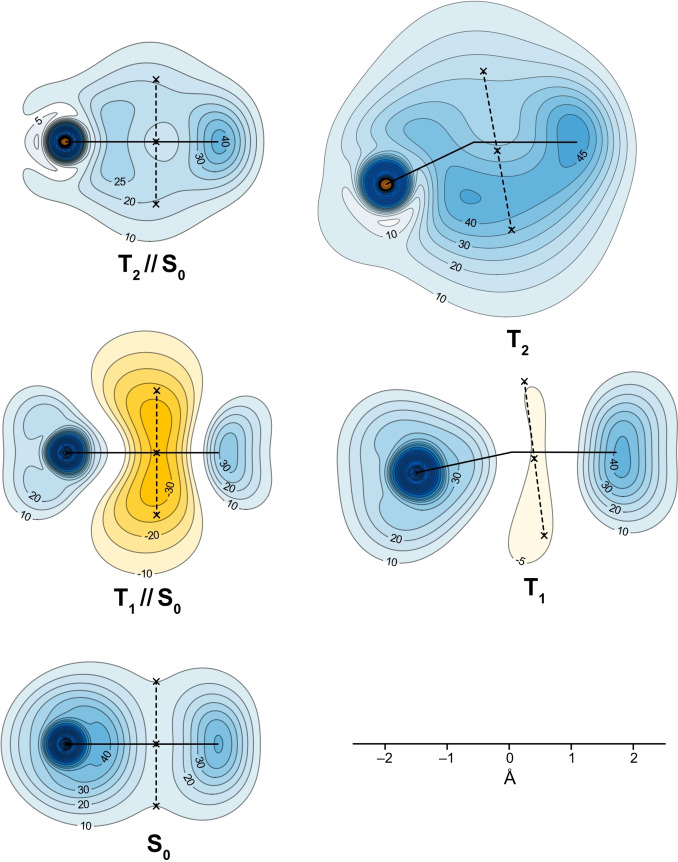
Isotropic shielding contour plots in the vertical symmetry planes passing through the sulfur atoms and the midpoints of the opposite carbon‐carbon bonds for the S_0_, T_1_ and T_2_ electronic states of thiophene. Contour levels requested at −30(5)−5, 10(5)50, 60(20)240 ppm, orange (deshielded) to blue (shielded). Solid black lines show the projections of the carbon‐sulfur and carbon‐carbon bonds. The positions of NICS(0) and NICS(±1) are marked by crosses and connected by dashed lines.

Isotropic shielding isosurfaces for the S_0_, vertical T_1_ and T_2_ (T_1_//S_0_ and T_2_//S_0_), and T_1_ and T_2_ electronic states of thiophene are shown in Figure 2. The isovalues of *σ*_iso_(**r**)=±16 ppm and the geometry orientations were chosen so as to highlight the details required for the current discussion. Further information about the behaviour of the isotropic shielding in the electronic states of thiophene studied in this paper is provided by a corresponding set of contour plots in the vertical symmetry planes passing through the sulfur atoms and the midpoints of the opposite carbon‐carbon bonds (Figure 3).

In line with previous studies of the *σ*_iso_(**r**) variations in the electronic ground state of thiophene,[^22^, ^47^] we observe that the five‐membered ring is very well‐shielded, even more so than the six‐membered ring in the electronic ground state of benzene[^16^, ^48^]; this can be attributed mainly to placing the 6 π electrons in a ring of a smaller size and suggests strong bonding interactions and aromatic stability. The shielding picture in the vertical T_1_ (T_1_//S_0_) state of thiophene includes a strongly deshielded dumbbell‐shaped region in the centre of the molecule. This deshielded region is smaller in size than the corresponding central deshielded region in the vertical T_1_ state of benzene^[16]^; in addition, it has been shown^[21]^ that UB3LYP‐GIAO overestimates the antiaromaticity of the vertical T_1_ state in comparison to CASSCF‐GIAO, and a switch to a CASSCF‐GIAO description of this state in thiophene is likely to suggest a lower level of antiaromaticity. It should be mentioned that the S_0_ geometry of thiophene has a lower symmetry (*C*_2v_) than that of S_0_ benzene (*D*_6h_) which can help the vertical T_1_ state of thiophene alleviate its antiaromatic character electronically, without geometry relaxation, in contrast to the vertical T_1_ state of benzene which needs geometry relaxation to alleviate its antiaromatic character. These observations indicate that the “amplitude” of the aromaticity reversal between the S_0_ and vertical T_1_ states of thiophene is smaller than that between the corresponding states of benzene; whereas the levels of aromaticity of the S_0_ states of benzene and thiophene are not that far off, the vertical T_1_ state of benzene is more antiaromatic than the vertical T_1_ state of thiophene.

The comparison between the shielding picture for the vertical T_1_ state and that at the optimized geometry of this state shows that as a result of the geometry relaxation the size of the central deshielded region is reduced considerably and it no longer stretches to the *σ*_iso_(**r**)=−16 ppm isosurface; this is accompanied by an increase in the shielding over all bonds, but not to the extent observed in the electronic ground state. These changes illustrate the extent to which the T_1_ geometry optimization allows the molecular framework to counteract the antiaromatic character of this state and decrease its level of antiaromaticity. It should be noted that, despite the use of larger basis sets, the S_0_ and T_1_ shielding isosurfaces shown in Figure 2 are visually indistinguishable (except for the change in orientation) from the corresponding isosurfaces reported in ref. [22].

As it was mentioned in the Introduction, a common feature of the shielding pictures observed in the vertical S_2_ states of benzene,^[16]^ regular octagonal cyclooctatetraene^[20]^ and naphthalene,^[21]^ and in the vertical S_1_ state of anthracene^[21]^ is that the ring interiors are well‐shielded all over, but the carbon‐hydrogen bonds are less shielded than in the corresponding S_0_ states. The whole interior of the thiophene ring in its vertical T_2_ state is also well‐shielded, which indicates that this is the first example of a “strongly aromatic” triplet state observed in the literature. The presence of the sulfur atom leads to certain differences in the shielding over bonds: In the vertical T_2_ state of thiophene the C_3_−H and C_4_−H bonds are less shielded than in the S_0_ state, but the C_2_−H and C_5_−H bonds are more shielded than in the S_0_ state; in addition, the carbon‐sulfur bonds are significantly less shielded than in the S_0_ state.

At the optimized geometry of the T_2_ state we observe not only a carbon‐carbon bond alternation pattern similar to that in the S_0_ state (see above) but also a significant increase of the shielding within the ring interior (compare the T_2_//S_0_ and T_2_ isotropic shielding contour plots in Figure 3). This is an indication that, despite making the molecule non‐planar, the relaxation of the geometry of the T_2_ state of thiophene enhances its aromaticity. The puckering of the ring in the optimized T_2_ geometry increases the shielding over the carbon‐sulfur bonds but the immediate surroundings of the sulfur nucleus remain deshielded (Figure 3).

The carbon atoms in the S_0_, T_1_ and T_2_ electronic states of thiophene are surrounded by small ovoid deshielded regions inside which *σ*_iso_(**r**) first becomes negative and then switches back to positive close to the nucleus. These regions are easier to notice in the *σ*_iso_(**r**) isosurface plots for the T_1_ state in which there is less shielding over the carbon‐carbon and carbon‐sulfur bonds. Similar deshielded ‘halos’ around sp^2^ and sp hybridized carbon atoms and other sp^2^ hybridized first main row atoms have been observed previously in conjugated rings,[^16^, ^47^, ^49^] as well as in open‐chain conjugated molecules such as ethene, ethyne and *s*‐*trans*‐1,3‐butadiene.[^50^, ^51^] The deshielded ‘halos’ around the carbon atoms in all three electronic states suggests that the hybridisation states of these atoms are close to sp^2^. There is no such ‘halo’ around the sulfur atom but in the T_2_ state, very close to the sulfur atom, *σ*_iso_(**r**) switches from positive to negative and remains negative at the atomic position (see Figure 3 and Table 4). This sign change is supported by the Mulliken populations of the sulfur atom, −0.277, −0.290 and 0.211 in S_0_, T_1_ and T_2_, respectively (calculated at the same level of theory as the data in Table 4).

**Table 4:** NICS values and isotropic nuclear shieldings for the symmetry‐unique nuclei in the S_0_, T_1_ and T_2_ electronic states of thiophene (in ppm). The NICS(+1) values correspond to positions above the ring (Figure 3). The hydrogen atoms are attached to the respective carbon atoms from the *σ*_iso_(^13^C) column.

State	NICS(0)	NICS(±1)	*σ*_iso_(^33^S)	*σ*_iso_(^13^C)	*σ*_iso_(^1^H)
S_0_	−12.5	−10.0	231.3	50.3 (C_2_) 53.0 (C_3_)	24.3 24.5
T_1_//S_0_	31.1	27.3	320.3	80.0 (C_2_) 36.2 (C_3_)	28.7 26.9
T_1_	5.4	4.8 (+1) 5.8 (−1)	309.5	69.6 (C_2_) 47.8 (C_3_)	26.9 25.7
T_2_//S_0_	−18.0	−19.2	−158.3	136.1 (C_2_) 50.7 (C_3_)	27.3 23.3
T_2_	−31.4	−23.5 (+1) −36.1 (−1)	−190.4	115.8 (C_2_) 50.4 (C_3_))	24.9 22.8

The NICS values and sulfur, carbon and proton isotropic shieldings for the S_0_, T_1_ and T_2_ electronic states of thiophene are collected in Table 4. The NICS(0) and NICS(1) values at the S_0_ geometry and the NICS(0) and NICS(±1) values at the T_1_ and T_2_ geometries support the conclusions following from the comparisons between the *σ*_iso_(**r**) variations illustrated by Figures 2 and 3. However, further examination of the contour plots in Figure 3 shows that although the sign of *σ*_iso_(**r**) remains the same at points reasonably close to the NICS(0) and NICS(±1) positions, its magnitude can change by more than 5 ppm. This behaviour is an indication that while NICS values can be useful as qualitative aromaticity criteria, the somewhat arbitrary choices of the positions at which these values are calculated restricts their utility as quantitative measures of aromaticity, especially for non‐planar rings. The *σ*_iso_(**r**) volume data used to construct the isosurface and contour plots in Figures 2 and 3, respectively, is free of this position‐choice bias and, at nuclear positions, become the nuclear isotropic shieldings which can be compared to experimental gas‐phase and solution NMR measurements.

The *σ*_iso_(^33^S) and *σ*_iso_(^13^C) values vary considerably between the S_0_, T_1_ and T_2_ states. However, the S_0_ *σ*_iso_(^33^S), *σ*_iso_(^13^C) and *σ*_iso_(^1^H) values are in reasonable agreement with the results of other DFT calculations^[52]^ which suggests that the nuclear shielding results for all three states are representative of what can be obtained using DFT and UDFT calculations. As expected for a switch from an aromatic to an antiaromatic state, the proton shieldings increase on passing from S_0_ to the vertical T_1_ state and then decrease as the level of antiaromaticity is reduced at the optimized T_1_ geometry. Passing from the antiaromatic vertical T_1_ state to the aromatic vertical T_2_ state is accompanied by a decrease in the proton shieldings; these shieldings decrease further at the optimized T_2_ geometry, highlighting the increase in aromaticity.

Spin density isosurfaces for the vertical T_1_ and T_2_ (T_1_//S_0_ and T_2_//S_0_), and T_1_ and T_2_ electronic states of thiophene obtained from triplet UB3LYP/def2‐TZVPPD calculations are shown in Figure S1. While there are differences between the shapes of the spin density distributions for the T_1_ and T_2_ states, there appears to be no obvious connection between these differences and the aromaticity reversal between the two states.

## Conclusions

The magnetic properties of the electronic ground state and the two lowest triplet states of thiophene, all of which are accessible through DFT or UDFT calculations, strongly suggest that thiophene which is aromatic in its electronic ground state becomes antiaromatic in its lowest vertical triplet state and then reverts to being aromatic in its second vertical triplet state. The relaxation of the geometry through geometry optimization is found to have an opposite effect in the two lowest triplet states: The antiaromaticity of the lowest triplet state is reduced whereas the aromaticity of the second triplet state is enhanced.

The off‐nucleus isotropic shielding picture around the T_2_ state of thiophene closely resembles those observed in the vertical S_2_ states of benzene,^[16]^ regular octagonal cyclooctatetraene^[20]^ and naphthalene,^[21]^ and in the vertical S_1_ state of anthracene,^[21]^ all of which can be thought to be “strongly aromatic” because of exhibiting well‐shielded ring interiors and very negative NICS values. However, the comparison between the optimized geometries of the S_0_ and T_2_ states of thiophene does not suggest that T_2_ is the more aromatic state: Whereas the difference between the lengths of the carbon‐carbon “single” and “double” bonds is slightly smaller in T_2_, the geometry of this state is non‐planar and the carbon‐sulfur bonds are longer which restricts the ability of the sulfur atom to contribute to the conjugated system. In addition, the lower shielding over the carbon‐sulfur bonds in the T_2_ state can be considered as a destabilizing and aromaticity‐reducing factor. These observations are supported by the lower HOMA value for the T_2_ state.

The changes in the NICS values between the vertical T_1_ and T_2_ states and the respective states at their optimised geometries show that NICS values correctly follow the changes in aromaticity along the potential energy surface of a single electronic state. However, the use of NICS values for the purpose of quantitative comparisons between the aromaticities of different electronic states cannot be justified theoretically and should be avoided. Thus, while we can add the T_2_ state of thiophene to the list of “strongly aromatic” low‐lying excited states observed previously, all of which were singlet, the current results suggest that these states should be referred to simply as “aromatic” because an assertion that an excited state exhibiting very negative NICS values is more aromatic than the corresponding electronic ground state cannot be justified through a careful comparison between the energies, geometries (for non‐vertical excitations) and off‐nucleus shielding pictures of the two states. For thiophene, the simple HOMA criterion offers a more realistic assessment of the relative aromaticities of the S_0_ and T_2_ states.

As research into excited state aromaticity expands to include excited states other than S_1_ and T_1_, it is very likely that it will come across a number of further examples of states with very negative NICS values. One way to properly assess the aromaticity levels of such states is to follow the procedures outlined in the current study of the aromaticity reversals between the S_0_, T_1_ and T_2_ states of thiophene.

## Supporting Information Summary

Gaussian cube files with isotropic shielding values; detailed optimized geometries of the S_0_, T_1_ and T_2_ states of thiophene, additional computational details, cartesian coordinates and other computational data.

## Conflict of Interests

The authors declare no conflict of interest.

1

## Supporting information

As a service to our authors and readers, this journal provides supporting information supplied by the authors. Such materials are peer reviewed and may be re‐organized for online delivery, but are not copy‐edited or typeset. Technical support issues arising from supporting information (other than missing files) should be addressed to the authors.

Supporting Information

## Data Availability

The data that support the findings of this study are available in the supplementary material of this article.
