# Supplementary material for: Aromaticity and Antiaromaticity Reversals between the Electronic Ground State and the Two Lowest Triplet States of Thiophene
Source: Chemphyschem. 2024 Nov 5;26(1):e202400758. doi: 10.1002/cphc.202400758 (PMC11747585; doi:10.1002/cphc.202400758)
Supplement: Supplementary file 1 — Supporting Information [file CPHC-26-e202400758-s001.pdf]

ChemPhysChem

Supporting Information

Aromaticity and Antiaromaticity Reversals between the Electronic Ground State and the Two Lowest Triplet States of Thiophene

Edward Cummings and Peter B. Karadakov*

Supporting Information

Aromaticity Reversals between the Electronic Ground State and the Two Lowest Triplet States of Thiophene

Edward Cummings and Peter B. Karadakov*
Department of Chemistry, University of York, Heslington, York, YO10 5DD, U.K.

E-mail: peter.karadakov@york.ac.uk

Table of Contents

1. Gaussian Cube Files with Isotropic Shielding Values __________________S2
2. Detailed Optimized Geometries of the S_0, T_1 and T_2 States of Thiophene _S2
3. Additional Computational Details____________________________________S3
4. Spin Density Isosurfaces for the T_1 and T_2 States of Thiophene_________S4
5. Cartesian Coordinates and Other Computational Data _________________S4

1. Gaussian Cube Files with Isotropic Shielding Values

A zip archive of Gaussian cube files with isotropic shielding values for the S_0, vertical T_1 (T_1//S_0), vertical T_2 (T_2//S_0), T_1 and T_2 electronic states of thiophene is available as a separate download.

2. Detailed Optimized Geometries of the S_0, T_1 and T_2 States of Thiophene

Table S1. Comparison between selected structural parameters from the S_0 B3LYP-D3(BJ)/def2-TZVP and B3LYP-D3(BJ)/def2-TZVPPD optimized geometries of thiophene and those from a coupled-cluster calculation with single, double, and perturbative triple excitations in the cc-pCV5Z basis set [CCSD(T)/cc-pCV5Z],[19] and from a precise semi-experimental equilibrium structure (SE)[19] (for the atom numbering scheme, see Figure 1).

	S_0 RB3LYP^a	S_0 RB3LYP^b	S_0 CCSD(T)	S_0 SE
$R(S_1–C_2)$	1.71823	1.71845	1.70944	1.71049 (18)
$R(C_2–C_3)$	1.36512	1.36533	1.36632	1.36564 (31)
$R(C_3–C_4)$	1.42227	1.42239	1.42216	1.4224 (10)
$R(C_2–H)$	1.07737	1.07665	1.07633	1.07714 (17)
$R(C_3–H)$	1.08031	1.07962	1.07860	1.07856 (14)
$\angle(C_2–S_1–C_5)$	91.891	91.896	92.101	92.047 (15)
$\angle(S_1–C_2–C_3)$	111.495	111.489	111.596	111.608 (16)
$\angle(S_1–C_2–H)$	120.292	120.275	120.286	120.065 (28)
$\angle(C_2–C_3–H)$	123.418	123.390	123.416	123.414 (23)

^{a}Results obtained with the def2-TZVP basis set.
^{b}Results obtained with the def2-TZVPPD basis set.

Table S2. Comparison between selected structural parameters from the T_1 UB3LYP-D3(BJ) and TDA-B3LYP-D3(BJ) geometries of thiophene optimized using the def2-TZVP and def2-TZVPPD basis sets (for the atom numbering scheme, see Figure 1).

	T_1 UB3LYP^a	T_1 UB3LYP^b	T_1 TDA-B3LYP^a	T_1 TDA-B3LYP^b
$R(S_1–C_2)$	1.77092	1.77073	1.76978	1.76971
$R(C_2–C_3)$	1.46337	1.46310	1.45872	1.45838
$R(C_3–C_4)$	1.34309	1.34348	1.34770	1.34816
$R(C_2–H)$	1.08176	1.08077	1.08076	1.07976
$R(C_3–H)$	1.08083	1.08010	1.08049	1.07976
$\angle(C_2–S_1–C_5)$	89.603	89.549	89.406	89.346
$\angle(S_1–C_2–C_3)$	111.114	111.141	111.534	111.572
$\angle(S_1–C_2–H)$	120.104	120.190	120.193	120.275
$\angle(C_2–C_3–H)$	121.800	121.838	121.876	121.911
$\angle(S_1–C_1–C_2–C_3)$	9.163	9.343	8.236	8.386

∠(C_4–C_3–C_2–H)	−146.691	−147.333	−150.368	−151.213
∠(C_5–C_4–C_3–H)	−178.935	−178.833	−178.794	−178.708

[a]Results obtained with the def2-TZVP basis set.
[b]Results obtained with the def2-TZVPPD basis set.

Table S3. Comparison between selected structural parameters from the T_2 UB3LYP-D3(BJ) and TDA-B3LYP-D3(BJ) geometries of thiophene optimized using the def2-TZVP and def2-TZVPPD basis sets (for the atom numbering scheme, see Figure 1).

	T_2 UB3LYP[a]	T_2 UB3LYP[b]	T_2 TDA-B3LYP[a]	T_2 TDA-B3LYP[b]
R(S_1–C_2)	1.76838	1.76627	1.77144	1.76917
R(C_2–C_3)	1.40098	1.40060	1.39813	1.39780
R(C_3–C_4)	1.43410	1.43506	1.43778	1.43872
R(C_2–H)	1.07698	1.07603	1.07704	1.07604
R(C_3–H)	1.08081	1.08009	1.08081	1.08009
∠(C_2–S_1–C_5)	93.550	93.513	93.338	93.304
∠(S_1–C_2–C_3)	105.604	105.559	105.561	105.541
∠(S_1–C_2–H)	117.899	118.040	117.884	118.060
∠(C_2–C_3–H)	123.107	123.187	123.204	123.282
∠(S_1–C_1–C_2–C_3)	17.558	17.941	17.999	18.324
∠(C_4–C_3–C_2–H)	162.033	162.824	162.137	163.034
∠(C_5–C_4–C_3–H)	−178.378	−178.331	−178.572	−178.484

[a]Results obtained with the def2-TZVP basis set.
[b]Results obtained with the def2-TZVPPD basis set.

3. Additional Computational Details

All B3LYP calculations reported in this paper were carried out using the GAUSSIAN "SuperFine" integration grid. All geometry optimizations included Grimme's D3 empirical dispersion corrections with Becke–Johnson damping, were carried out under the GAUSSIAN "VeryTight" convergence criteria, and were combined with analytical harmonic frequency calculations. The TD-B3LYP and TDA-B3LYP calculations were carried out for triplet states only, solving for 10 triplet states.

Points making up the grids of isotropic magnetic shielding values and/or corresponding to NICS positions were specified in the GAUSSIAN input files as ghost atoms without basis functions (symbol "Bq"). To improve accuracy, the GAUSSIAN "CPHF(Separate)" option was used in all NMR calculations, but computational experience indicates that, even with this option, accuracy remains acceptable only as long as the number of atoms and ghost atoms included in a single calculation does not exceed about 250. As a consequence, collecting all data

for a grid requires running a large number of separate GAUSSIAN calculations. To prepare the set of input files, use was made of a purpose-written program which is modified to include the GAUSSIAN route section, geometry and grid specification and for each molecule.

4. Spin Density Isosurfaces for the T_1 and T_2 States of Thiophene

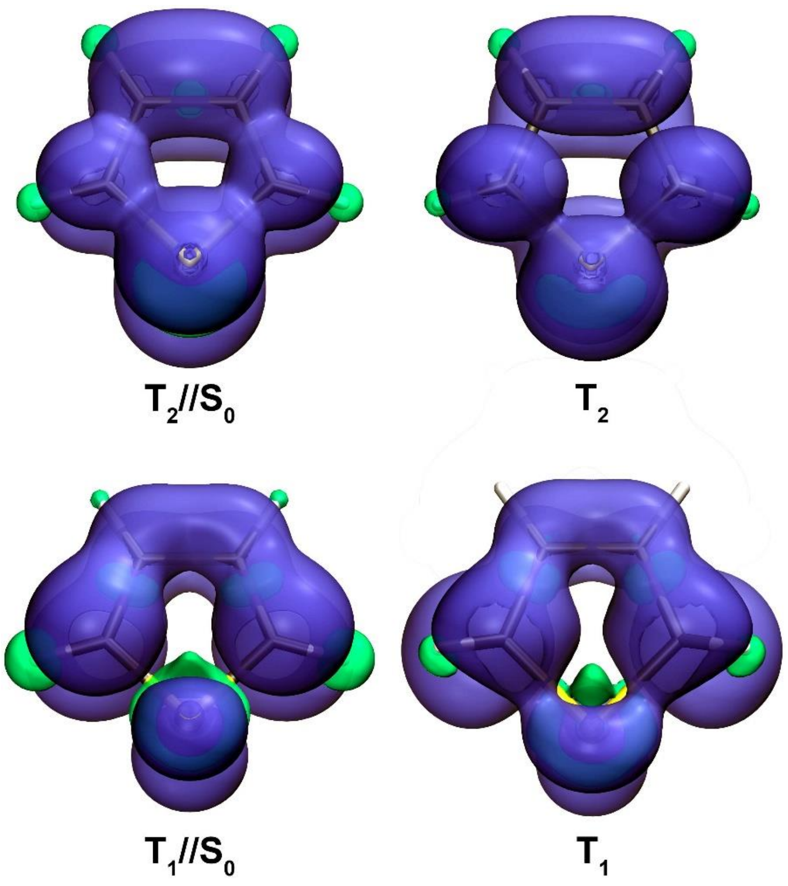

Figure S1. Spin density isosurfaces at $\rho_\alpha(\mathbf{r}) - \rho_\beta(\mathbf{r}) = \pm0.002$ a.u. ("+" in violet, "–" in green) for the T_1 and T_2 electronic states of thiophene. $\rho_\alpha(\mathbf{r})$ and $\rho_\beta(\mathbf{r})$ stand for the α and β spin electron densities. The $T_1//S_0$ and $T_2//S_0$ isosurfaces correspond to the $S_0 \rightarrow T_1$ and $S_0 \rightarrow T_2$ vertical excitations, respectively. Triplet UB3LYP/def2-TZVPP results at the S_0 B3LYP-D3(BJ)/def2-TZVPP and T_1 or T_2 UB3LYP-D3(BJ)/def2-TZVPP optimized geometries.

5. Cartesian Coordinates and Other Computational Data

The geometries at which NICS were calculated include the coordinates of the respective ghost atoms. All coordinates are given in Å.

RB3LYP-D3(BJ)/def2-TZVPPD total energy *E*, lowest vibrational frequency ν and optimized geometry of the S_0 state of thiophene, local minimum of C_{2v} symmetry.

E = –553.116264 Ha; ν = 464.6 cm^{-1} (B_1).

```
C   0. -1.23506474    0.0029745742
```

```
C  0. -0.7111951214  1.2638048817
C  0.  0.7111951214  1.2638048817
C  0.  1.23506474    0.0029745742
S  0.  0.           -1.1918798942
H  0. -2.2716871604 -0.2878728051
H  0. -1.3156474913  2.1583573163
H  0.  1.3156474913  2.1583573163
H  0.  2.2716871604 -0.2878728051
Bq 0.  0.            0.268335804
Bq 1.  0.            0.268335804
```

UB3LYP-D3(BJ)/def2-TZVPPD total energy *E*, $<S^2>$ expectation value, lowest vibrational frequency ν and optimized geometry of the T_1 state of thiophene, local minimum of C_s symmetry.

E = –552.998697 Ha; $<S^2>$ = 2.0082; ν = 154.2 cm^{-1} (A″).

```
C  -0.0091933388  1.2471599657  0.0766216545
C   1.3360016454  0.6717395463  0.0730281208
C   1.3360016454 -0.6717395463  0.0730281208
C  -0.0091933388 -1.2471599657  0.0766216545
S  -1.2379868783  0.           -0.1882070473
H  -0.2741439796  2.1775016845  0.5586332505
H   2.2207106124  1.2911491076  0.0886824957
H   2.2207106124 -1.2911491076  0.0886824957
H  -0.2741439796 -2.1775016845  0.5586332505
Bq  0.283125947   0.           -0.005719842
Bq  0.411319869   0.           -0.997468963
Bq  0.154932024   0.            0.986029278
```

UB3LYP-D3(BJ)/def2-TZVPPD total energy *E*, $<S^2>$ expectation value, lowest vibrational frequency ν and optimized geometry of the T_2 state of thiophene, local minimum of C_s symmetry.

E = – 552.955038 Ha; $<S^2>$ = 2.0076; ν = 227.7 cm^{-1} (A′).

```
C  -0.0382150842  1.2866379572  0.1961473002
C   1.2415468761  0.7175304189  0.1960344652
C   1.2415468761 -0.7175304189  0.1960344652
C  -0.0382150842 -1.2866379572  0.1961473002
S  -1.1289315237  0.           -0.3278987619
H  -0.293024974   2.3006425353 -0.0582097954
H   2.1488929434  1.3028796178  0.2223849056
H   2.1488929434 -1.3028796178  0.2223849056
H  -0.293024974  -2.3006425353 -0.0582097954
Bq  0.255546412   0.            0.091292954
Bq  0.432517444   0.           -0.892923107
Bq  0.07857538    0.            1.075509014
```

TDA-B3LYP-D3(BJ)/def2-TZVPPD total energy *E*, lowest vibrational frequency ν and optimized geometry of the T_1 state of thiophene, local minimum of C_s symmetry.

E = –552.997199 Ha; ν = 201.4 cm^{-1} (A′).

```
C -0.005718859   1.2442120175  0.0697065553
C  1.3365915079  0.6740787907  0.0658540487
C  1.3365915079 -0.6740787907  0.0658540487
```

```
C -0.005718859  -1.2442120175  0.0697065553
S -1.24179289    0.           -0.1667651172
H -0.2701601     2.2000178124  0.4967754752
H  2.2202233954  1.2943836009  0.0832524834
H  2.2202233954 -1.2943836009  0.0832524834
H -0.2701601    -2.2000178124  0.4967754752
```

TDA-B3LYP-D3(BJ)/def2-TZVPPD total energy E, lowest vibrational frequency ν and optimized geometry of the T_2 state of thiophene, local minimum of C_s symmetry.

E = –552.956739 Ha; ν = 226.2 cm^{-1} (A′).

```
C -0.0380207267  1.2865390034  0.2002191847
C  1.2395331719  0.7193621402  0.2005698494
C  1.2395331719 -0.7193621402  0.2005698494
C -0.0380207267 -1.2865390034  0.2002191847
S -1.1276558016  0.           -0.3359483176
H -0.2908740151  2.3016767016 -0.0516050324
H  2.147500462   1.3038191172  0.2248491839
H  2.147500462  -1.3038191172  0.2248491839
H -0.2908740151 -2.3016767016 -0.0516050324
```
